# AI exposure and task detachability: migrant anchoring and work-linked health protection among highly educated foreign-born adults in the United States

**DOI:** 10.3389/fpubh.2026.1929386

**Published:** 2026-10-05

**Authors:** Tse Hou Hew, Rajah Rasiah, Nina Xie

**Affiliations:** 1 Asia-Europe Institute, Universiti Malaya, Kuala Lumpur, Malaysia; 2 Department of Management, Lingnan University, Hong Kong, Hong Kong SAR, China

**Keywords:** artificial intelligence, health coverage, material security, migrant workers, occupational epidemiology, occupational exposure assessment, social-reproductive anchoring, task detachability

## Abstract

**Background:**

Occupation-level AI exposure indices describe the potential overlap between occupational work content and AI capabilities rather than individual AI use or employer adoption. However, task organisation and access to work-linked protections may differ across occupational contexts. We examined whether associations between a compound high-AIOE/high-task-detachability occupational profile and upstream insurance and material conditions varied by migrant anchoring among highly educated foreign-born adults in the United States.

**Methods:**

We linked 2017–2024 IPUMS American Community Survey (ACS) data for adults aged 25–64 with a bachelor’s degree or higher to AI Occupational Exposure (AIOE) and O*NET 30.3 measures. High AIOE was defined as the top occupational quartile. The focal profile combined high AIOE with high task detachability and was compared with lower-AIOE occupations. Migrant anchoring combined citizenship, US residence duration, English proficiency, family co-residence, and homeownership; scores of 0–2 indicated lower anchoring. The three principal domain-specific outcomes were absence of employer- or union-linked health coverage from any eligible source among employed wage and salary workers, uninsurance, and family income below 200% of the federal poverty threshold. All-adult work-linked Social Reproduction Instability (W-SRI) ≥ 2 was retained as the focal cumulative summary measure. Weighted models were adjusted for demographic, regional, annual, and broad occupational characteristics. Inference used CR2/Satterthwaite covariance, wild cluster bootstrap tests, and leave-one-cluster-out analyses.

**Results:**

The analysis included 746,995 foreign-born person-year observations, of which 22.47% were classified as having lower anchoring. Among adults with higher anchoring, the focal occupational profile and lower-AIOE occupations showed few differences in the four principal outcomes. Among adults with lower anchoring, the focal occupational profile was associated with a lower prevalence of all four adverse outcomes. The corresponding between-stratum differences were −10.68 percentage points for absence of employer- or union-linked coverage, −6.75 percentage points for uninsurance, −7.90 percentage points for poverty, and −7.55 percentage points for cumulative burden. Magnitudes remained similar after excluding 2020, restricting the sample to 2018–2024, adding state fixed effects, and restricting the sample to currently employed respondents. In the labour force-restricted analysis, the unemployment modification was −0.37 percentage points (95% CI −1.15 to +0.42) and was imprecisely estimated.

**Conclusion:**

Among highly educated foreign-born adults with lower anchoring, the compound occupational profile was associated with a lower prevalence of coverage absence, uninsurance, poverty, and cumulative burden than occupations with lower AIOE. The findings describe occupation-level differences in upstream insurance and material conditions rather than causal effects of AI adoption or task detachability.

## Introduction

Artificial intelligence and digitalisation are increasingly relevant to public health because they may reorganise tasks, supervision, pace, autonomy and access to employment-related resources without eliminating an occupation (1–5). Occupation-level AI indices help locate where technological capabilities overlap with work content, but they measure potential exposure rather than individual use, employer adoption, realised automation, or post-adoption health effects (6–11). Here, work-linked protection refers to insurance and material resources connected to occupational and employment arrangements. These resources matter to public health because they shape access to care, financial resilience, and the capacity to absorb job or household transitions.

In the United States, health insurance access and material security remain unevenly connected to occupation, work status, household coverage, and establishment characteristics (12–16). These pathways are consequential for foreign-born workers, whose occupational placement can reflect legal and recruitment routes, language, residence duration, credential recognition, racialisation, and family resources (17–25). A bachelor’s degree does not eliminate this heterogeneity. Employment precarity and access to protection also vary across places, making geographic context relevant even when the study is not designed as a spatial analysis (15, 16, 25–27). Highly educated migrants may occupy substantially different labour-market positions despite having similar formal qualifications. Some enter professional or technical roles through employer recruitment and internationally recognised credentials, whereas others experience skill discounting, restricted mobility, or weaker access to benefit-bearing employment (18, 19, 24, 28, 29). Migrant anchoring captures a related but distinct dimension: the legal, temporal, language, family, and housing resources that connect workers to destination-country institutions (17–23). Lower anchoring may indicate fewer settlement resources, but it may also characterise recent professional migrants who have entered advantaged occupational channels before acquiring citizenship, establishing long residence, or becoming homeowners.

These research areas have largely developed separately: AI exposure research maps potential task overlap, occupational health research examines work design, and migration/precarity research examines occupational sorting and access to protection. Less is known about how a compound high-AI Occupational Exposure (AIOE)/high-task-detachability occupational profile is associated with upstream insurance and material conditions among highly educated foreign-born adults, or whether these associations vary across settlement resource profiles. We therefore ask the following questions: (RQ1) How is membership in this compound occupational profile associated with employer- or union-linked coverage access, uninsurance, poverty, and cumulative burden relative to lower-AIOE occupations? (RQ2) Do these associations differ between lower- and higher-anchoring groups? The study contributes an occupation-level framework that integrates AI exposure with task organisation, examines insurance and material conditions as upstream public health outcomes, and evaluates heterogeneity across migrant anchoring strata and geographic specifications.

## Conceptual and empirical background

### Occupational AI exposure as potential exposure

Occupational AI exposure indices describe relative technological relevance at the level of occupational work content. AIOE links AI applications to the abilities required in O*NET occupations and estimates potential occupational exposure; by design, it does not establish that an employer has adopted AI or that AI complemented or substituted for labour in a particular job (11). Newer task-based measures similarly estimate which tasks could be affected under specified technological assumptions and do not predict adoption timelines (9). Accordingly, AIOE was used as an occupation-level indicator of potential technological relevance. The broader automation literature distinguishes predicted occupational susceptibility from realised labour market effects (30–32). In this study, AIOE identifies potential technological overlap, whereas detachability describes how readily work can be separated from a fixed place, embodied activity, or face-to-face interaction.

### Task organisation, digital work, and occupational health

Potential exposure becomes relevant to occupational health through the organisation of work rather than through technology alone. Digital systems can expand flexibility, feedback, and autonomy, but they can also increase monitoring, workload, work–home boundary permeability, and reduced decision authority; their consequences depend on sociotechnical implementation and the balance of job demands and resources (1–5, 33). Evidence that occupational standing and the organisation of essential work differentiated work-related infectious exposure during the COVID-19 pandemic further illustrates why task and organisational context matter for worker health (34, 35). Occupational task data can identify whether work is structurally compatible with remote performance, but such feasibility remains distinct from actual remote-work uptake (36). Evidence on telework and employee health is heterogeneous and generally of low certainty (37). In practical terms, higher task detachability describes occupations dominated by digital, abstract, or remotely tradable work with less dependence on physical presence, equipment, or face-to-face interaction. Task detachability is therefore used here as an occupation-level contextual feature of the focal high-AIOE profile, not as a measure of individual technology use or as a health intervention.

### Precarious employment, work-linked protection, and migrant occupational sorting

Precarious employment combines insecurity in income, hours worked, continuity, rights, and access to employment-related protection and is increasingly treated as an upstream determinant of health (13, 15). Evidence also links both job insecurity and unemployment to adverse health outcomes, although the mechanisms differ across employment contexts (38). In the United States, medical benefits vary by occupation, work status, wages, bargaining coverage, and establishment characteristics (16). For immigrants, lower employer-sponsored coverage has been traced largely to whether workers are employed by firms that offer coverage, rather than only to whether workers enrol after an offer is made (12). The American Community Survey (ACS) measure used in this study identifies access to employer- or union-linked coverage but not the identity of the policyholder, so coverage can derive from the respondent or another eligible family member. Degree attainment also does not eliminate occupational sorting: Census-based evidence documents substantial cross-origin variation in the placement of highly educated immigrants in skilled occupations (24). These studies motivate examining occupational placement and work-linked protection jointly, without treating degree status or high occupational AI exposure as evidence of realised advantage.

### Regional and spatial unevenness of precarity

Employment precarity is spatially uneven. Russian regional data show spatial clustering and spillovers in informal employment (26), while an EU NUTS-2 composite index identifies persistent core–periphery and crisis-related differences in flexible and precarious labour (27). Together, these studies show that labour market institutions, employment quality, and insurance access vary geographically (15, 16, 25–27). US evidence likewise shows regional variation in multidimensional employment precarity, with higher mean levels of precarity among residents of the South (15). Employer medical benefit access varies across worker, establishment, and census area characteristics (16), while neighbourhood disadvantage illustrates geographic variation below the level of broad census regions (39). A recent ACS study of Black immigrant adults further links insurance variation to citizenship, race/ethnicity, and the state Medicaid expansion context (25). Place is therefore treated as a labour market and policy context. The analysis adjusted for census region and evaluated state fixed effects to account for broad geographic variation.

## Materials and methods

### Study design and data

We used a repeated cross-sectional occupational population health design, pooling the 2017–2024 one-year American Community Survey samples obtained through IPUMS USA (40). The ACS does not follow the same respondents over time; therefore, estimates compare occupational groups across repeated annual samples rather than within-person change. The 2020 ACS one-year file used experimental weights after pandemic-related disruptions to data collection (41); a sensitivity analysis therefore excluded 2020.

The source file contained 4,789,999 unweighted person-years for adults aged 25–64 with a bachelor’s degree or higher, representing 495,586,705 weighted person-years. Occupation records were linked to AIOE and O*NET using reported OCCSOC codes. The ACS used a 2010 Standard Occupational Classification (SOC)-based code in 2017 and SOC-based codes from 2018 onwards. Exact six-digit matches were used where available; unresolved codes were linked through documented five-, four-, three- or two-digit prefix rules, using the unweighted arithmetic mean of source occupations sharing the prefix. As the pooled crosswalk did not encode year-specific SOC vintages, a 2018–2024 restriction was evaluated as a targeted sensitivity analysis. The linked sample contained 4,488,242 person-years, representing 464,719,960 weighted person-years. Primary interaction models were restricted to 746,995 linked foreign-born person-years, representing 83,420,333 weighted person-years, with valid social reproductive anchoring (SRA) and covariate information. The restriction to adults with a bachelor’s degree or higher was intended to focus the analysis on professional, managerial, technical, and information processing occupations in which AI-related task exposure is especially salient. It does not imply that degree holders form a homogeneous or uniformly advantaged population.

### Occupation-level exposure assessment

AIOE values were obtained from the AIOE Data Appendix accompanying Felten et al. (11), which contains 774 occupation codes, titles, and exposure scores. The checksum of the frozen source file is reported in the reproducibility package. The 75th percentile of the occupation-level distribution was 1.014434, identifying 194 of the 774 occupations as high-AIOE occupations; occupations below this value formed the lower-AIOE reference group. The cutoff was calculated across source occupations without person-level weighting. A top tercile sensitivity definition used a source-occupation cutoff of 0.7093937333.

O*NET measures were drawn from database release 30.3 (May 2026) using O*NET-SOC 2019 aligned with the 2018 SOC (42). This fixed occupational task snapshot was applied retrospectively to the 2017–2024 ACS samples and was not interpreted as a time-varying task measurement. Detachability and shielding were calculated from standardised occupation-level AIOE and O*NET components. Shielding was defined as the mean of standardised interpersonal or client skills, manual or equipment skills, client- or public-facing work context, and physical or embodied work context. Detachability was defined as the mean of standardised data and software intensity, software development intensity, cloud or network software intensity, abstract technical skills, digital or text communication, and a remote tradability proxy, minus the shielding score. All components were standardised across the 774 source occupations before classification. Component identifiers, non-missing counts, complete-case rules, formulas, and standardisation choices are documented in the source-data package.

Among high-AIOE occupations with complete task scores, the median value was 0.745438 for detachability and −0.225300 for shielding. The validation typology contained four quadrants: high detachability/low shielding, high detachability/high shielding, low detachability/high shielding, and low detachability/low shielding. In total, four high-AIOE occupations lacked complete task scores and were retained separately. The high-detachability/low-shielding and high-detachability/high-shielding quadrants contained 67 and 28 source occupations, respectively. The primary exposure category comprised all 95 high-AIOE occupations with detachability scores above the median; shielding was examined as a secondary task-context dimension in the four-quadrant analyses. Lower-detachability/high-shielding, lower-detachability/low-shielding, and task-score-unavailable occupations remained separate categories (Figure 1). In substantive terms, the focal category captures occupations that combine high potential AI relevance with task structures that are relatively digital, abstract, and geographically detachable. Shielding was retained to assess whether interpersonal, embodied, or equipment-dependent requirements differentiated this broader high-detachability profile.

**Figure 1:**
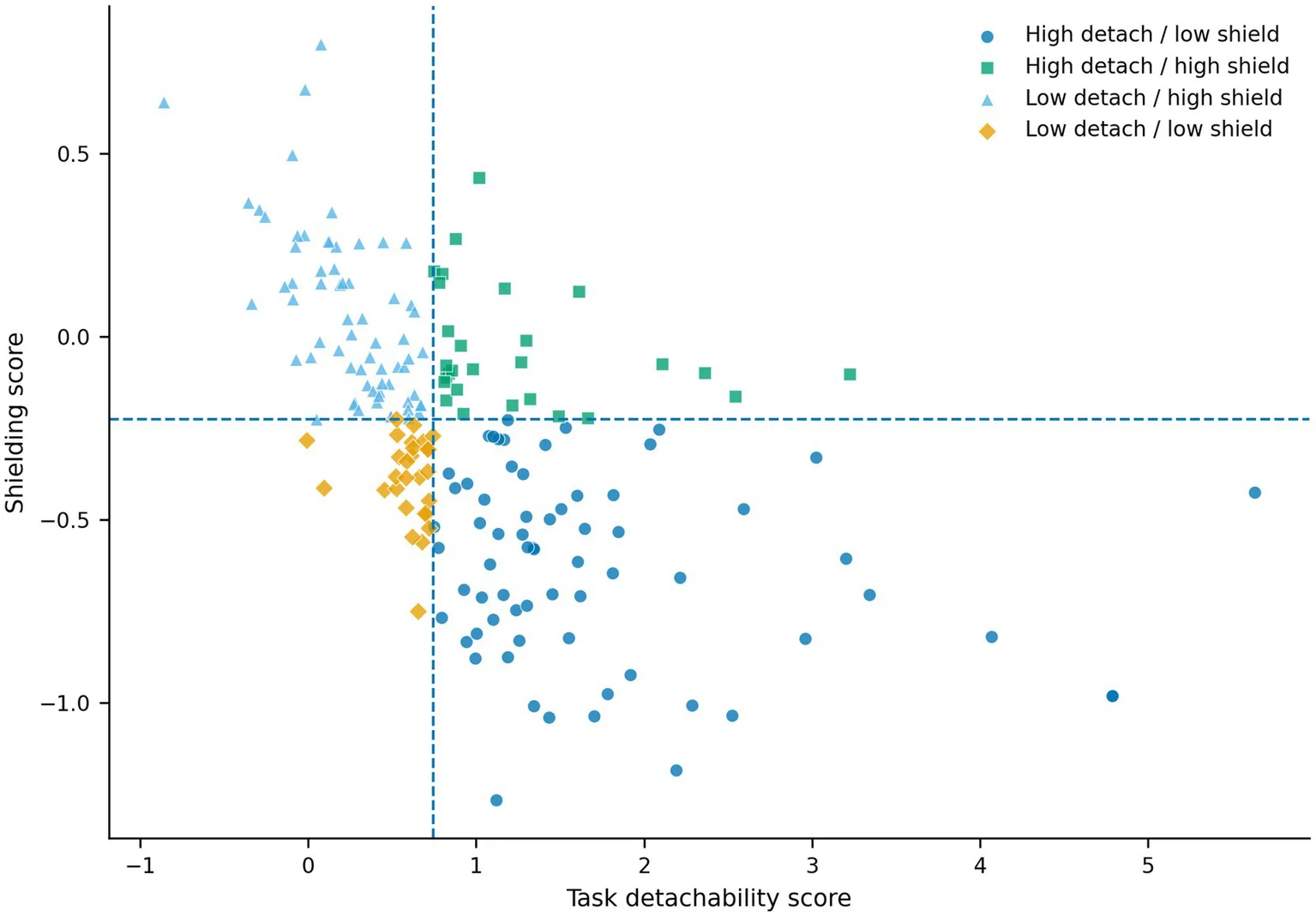
Task detachability and shielding among high-AIOE occupations.Note. Each point represents a source occupation with complete task scores (n = 190). Dashed lines indicate source-occupation medians used to define quadrants (detachability = 0.745438; shielding = −0.225300). The primary focal category combines the two high-detachability quadrants. Four high-AIOE source occupations lacking task scores are not plotted and remain separate in Table 1. AIOE and task scores are occupation-level measures and do not represent individual AI use.

The focal exposure contrast compared the compound high-AIOE/high-task-detachability occupational profile with lower-AIOE occupations. This contrast captures a compound occupational profile defined jointly by AIOE classification, task content, and detailed occupational composition. The focal profile was represented in nine two-digit SOC major groups and 92 linked OCCSOC patterns. Exact-match and prefix-match status were retained for sensitivity analysis. AIOE and task scores were interpreted as occupation-level indicators of potential technological relevance and task context.

### Social reproductive anchoring

SRA5 summed five contemporaneously observed domains among foreign-born respondents. Legal anchoring was defined by US citizenship; temporal anchoring by at least 10 years of US residence; language anchoring by speaking only English at home or speaking English very well or well; family anchoring by having a spouse and/or children in the household; and housing anchoring by homeownership. Scores ranged from 0 to 5; each component contributed one point. The resulting score records the number of anchoring resources observed at the time of the survey, rather than a latent trait or a temporally prior exposure. SRA5 scores of 0–2 were classified as lower anchoring, and scores of 3–5 as higher anchoring. Validation included component prevalence by exposure category and anchoring stratum, continuous and categorical SRA5 specifications, alternative lower-anchoring thresholds of 0–1 and 0–3, and leave-one-component-out scores. Each leave-one-component-out score was normalised to the proportion of the four retained domains present. These analyses evaluated sensitivity to the binary threshold, non-linearity, and the influence of individual anchoring components.

### Outcomes

The principal domain-specific outcomes were absence of employer- or union-linked health coverage, uninsurance, and family income below 200% of the federal poverty threshold. The coverage outcome was evaluated only among employed wage and salary workers. IPUMS HINSEMP identifies coverage through a current or former employer or union, including coverage obtained through another family member; it does not identify the policyholder or establish that coverage was supplied by the respondent’s current job (40). Uninsurance indicated no health insurance coverage from any source and was treated as a health security endpoint rather than as a direct measure of employer behaviour. POVERTY codes 1–199 were classified as below 200% of the federal poverty threshold, codes 200–501 as not below 200%, and code 000 or any invalid value were treated as missing.

Furthermore, two cumulative burden measures were kept distinct. The all-adult work-linked Social Reproduction Instability (W-SRI) marker was applied to all eligible adults and equalled one when at least two of four indicators were present: Uninsurance, non-employment, poverty below 200%, and any move during the previous year. Employer- or union-linked coverage was excluded from this composite to avoid counting non-employment twice. The employed-worker burden marker was limited to employed wage and salary workers and equalled one when at least two of three indicators were present: Absence of employer- or union-linked coverage, poverty below 200%, and a move during the previous year. The all-adult W-SRI measure was retained as the focal cumulative summary accompanying the three domain-specific outcomes, whereas the employed-worker burden measure was secondary. Neither measure was interpreted as a psychometric scale.

Secondary outcomes were unemployment among respondents in the labour force (LABFORCE = 2, with EMPSTAT = 2 defined as the event and EMPSTAT = 1 defined as the non-event), non-employment, CPI-adjusted real annual wage income among respondents with positive wages, within-state movement, and residence abroad one year earlier. Annual wages were expressed in 2024 dollars using CPI-U values (43) and modelled on the logarithmic scale. A log hourly wage proxy based on annual wage, weeks worked, and usual weekly hours was available consistently only for 2019–2024 and was therefore restricted to those years in a supplementary analysis.

### Primary covariates

Primary models included ACS year, age group, sex, race/ethnicity, educational attainment, US Census region, and two-digit SOC major-group fixed effects. These covariates accounted for differences in annual, demographic, regional, educational, and broad occupational composition. A targeted sensitivity analysis replaced region with state fixed effects. Industry, class of worker, weeks worked, hours worked, visa pathway, and employer characteristics were either not available or not included in the primary model; residual compositional differences are considered in the limitations.

### Statistical analysis

Person records were collapsed to non-identifying, weighted model cells defined by exposure category, SRA, year, age group, sex, race/ethnicity, education, region, SOC major group, and, where required, sensitivity variables. For each outcome, the cells retained the weighted denominator, weighted outcome total, and unweighted count. The aggregation retained the quantities required to reproduce the weighted models without redistributing individual IPUMS records. Binary outcomes were estimated using PERWT-weighted linear probability models, and wage outcomes were estimated using PERWT-weighted log-linear models. The primary model included an exposure category × lower-anchoring interaction, with lower-AIOE occupations and higher anchoring as the reference categories. For each exposure category, we estimated (1) the contrast with lower-AIOE occupations among higher-anchoring adults, (2) the corresponding contrast among lower-anchoring adults, and (3) the lower-minus-higher-anchoring difference between those contrasts. A negative occupational contrast indicates a lower prevalence of the adverse outcome in the focal profile than in lower-AIOE occupations. A negative between-stratum difference indicates that this focal profile contrast was more favourable among lower-anchoring than higher-anchoring adults. Binary results are reported in percentage points. Log wage contrasts were transformed as 100[exp(β) − 1] and are reported as percentage differences. Conventional cluster-robust covariance was calculated at the two-digit SOC major-group level for comparison. Primary inference used CR2-adjusted cluster-robust covariance and coefficient-specific Satterthwaite degrees of freedom because only 22 SOC major clusters were available and the focal exposure categories occupied fewer groups. These procedures allow the uncertainty to reflect the limited number of occupational clusters supporting the exposure, rather than the much larger number of individual person-year observations alone. Restricted null-imposed wild cluster bootstrap tests used 9,999 replications and Webb six-point weights for the high-detachability × lower-anchoring modification in the four principal outcomes. Leave-one-SOC-major-group-out analyses assessed influence and sign stability. The four primary CR2 interaction *p*-values were also evaluated using the Benjamini–Hochberg false discovery rate procedure.

### Sensitivity analyses

Exposure robustness analyses restricted the sample to exact six-digit SOC matches, defined high AIOE using the top tercile and recalculated task score medians within that set, compared the two high-detachability shielding quadrants, and estimated interactions involving standardised continuous AIOE, detachability, and shielding both separately and jointly. Correlations among the continuous scores were reported because conditional coefficients do not represent independent interventions.

Targeted temporal, geographic, and universe checks excluded the experimental 2020 ACS sample, restricted the analysis to 2018–2024, replaced census region with state fixed effects, combined state fixed effects with exclusion of 2020, and restricted analyses to respondents currently employed. POVERTY and household-based SRA components were defined using their valid ACS code ranges, with invalid and special codes treated as missing. Unemployment models were restricted to respondents in the labour force.

SRA sensitivity analyses used the alternative thresholds, continuous and factor specifications, and normalised leave-one-component-out scores described above. Analyses were implemented in Python 3.13.5 using DuckDB 1.5.4, pandas 2.2.3, NumPy 2.3.5, Patsy 1.0.2, and SciPy 1.17.0. No missing outcome was imputed. Each model used respondents with valid data for its outcome and covariates. ACS replicate weights were unavailable in the analytic file; variance estimates therefore accounted for occupational clustering but did not incorporate the full replicate-weight design.

## Results

### Analytic sample, exposure linkage, and anchoring

The linked foreign-born analytic sample included 746,995 person-year observations, representing 83,420,333 weighted observations (Table 1). Lower anchoring accounted for 22.47% of the weighted analytic sample, while high-detachability occupations represented 34.23% and were distributed across nine SOC major groups (Table 1). Weighted sample characteristics by anchoring stratum and SRA component composition by exposure category are reported in Supplementary Table S1, Panels A and B. SRA component composition differed across occupational contexts. Among lower-anchoring adults, 92.97% of those in high-detachability occupations met the language component, compared to 72.47% among those in lower-AIOE occupations. Citizenship, at least 10 years of residence, and homeownership were uncommon in both groups and were less prevalent in high-detachability occupations. Thus, the same SRA score reflected different combinations of language, legal, temporal, family, and housing resources across occupational groups (Supplementary Table S1, Panel B).

**Table 1:** Analytic sample and occupation-level exposure support.

Panel A. Analytic sample and exposure linkage			
Measure	Unweighted person-years	Weighted person-years	Weighted %
Total bachelor’s-degree-or-higher ACS sample, 2017–2024	4,789,999	495,586,705	100.00
Valid AIOE/O*NET-linked person-years	4,488,242	464,719,960	93.77
Exact six-digit SOC match	2,215,591	231,534,229	46.72
Prefix-based SOC match	2,272,651	233,185,731	47.05
Unmatched occupation exposure	301,757	30,866,745	6.23
Linked foreign-born analytic sample	746,995	83,420,333	17.95
Foreign-born higher anchoring (SRA5 3–5)	603,411	64,678,265	77.53
Foreign-born lower anchoring (SRA5 0–2)	143,584	18,742,068	22.47

Panel B. Primary Q75 exposure categories among linked foreign-born person-year observations							
Exposure category	Source occupations	Unweighted person-years	Weighted person-years	Weighted %	Lower anchoring within category, %	SOC major groups	Linked OCCSOC patterns
Lower AIOE	580	376,808	43,168,742	51.75	22.46	21	444
Focal high-AIOE/high-task-detachability profile	95	262,252	28,554,980	34.23	22.52	9	92
Low detachability/high shielding	67	93,443	10,115,468	12.13	22.93	10	44
Low detachability/low shielding	28	7,298	792,019	0.95	17.99	6	21
High AIOE/task score unavailable	4	7,194	789,124	0.95	19.58	3	5

The analytic population comprised adults aged 25–64 years with a bachelor’s degree or higher. Percentages for exact, prefix-based, and unmatched linkage use the full eligible sample; exposure percentages use the linked foreign-born analytic sample. High AIOE was defined at the source-occupation 75th percentile (1.014434). The within-category percentage is the weighted share classified as lower anchoring (SRA5 0–2).

### Primary protection and material security outcomes

Among higher-anchoring adults, the compound high-AIOE/high-task-detachability occupational profile and lower-AIOE occupations differed little in the prevalence of absence of employer- or union-linked coverage (−1.15 percentage points; 95% CI −5.15 to +2.85), uninsurance (+0.14; 95% CI −1.67 to +1.96), poverty below 200% (−0.18; 95% CI −2.94 to +2.58), or all-adult cumulative burden (+0.39; 95% CI −1.67 to +2.45). Among lower-anchoring adults, the focal profile had a lower prevalence of all four outcomes. The corresponding contrasts were −11.82 percentage points (95% CI −17.04 to −6.61), −6.60 (95% CI −9.94 to −3.27), −8.08 (95% CI −12.99 to −3.17), and −7.16 (95% CI −12.50 to −1.81), respectively (Table 2; Figure 2). This pattern can be interpreted in two steps. First, the focal profile and lower-AIOE occupations showed similar adjusted outcome levels among higher-anchoring adults. Second, the same occupational comparison was markedly more favourable among lower-anchoring adults, producing negative between-stratum estimates.

**Table 2:** Adjusted focal occupational profile contrasts by migrant anchoring for work-linked health protection and material security.

Panel A. Primary domain-specific outcomes and focal cumulative summary					
Outcome	Higher anchoring (SRA 3–5), difference (95% CI)	Lower anchoring (SRA 0–2), difference (95% CI)	Between-stratum difference (95% CI)	CR2 p (BH q)	Wild cluster bootstrap p
Absence of employer- or union-linked coverage	−1.15 (−5.15, +2.85)	−11.82 (−17.04, −6.61)	−10.68 (−15.88, −5.47)	0.0016 (0.0063)	0.0008
Uninsured	+0.14 (−1.67, +1.96)	−6.60 (−9.94, −3.27)	−6.75 (−10.57, −2.92)	0.0039 (0.0077)	0.0006
Poverty below 200%	−0.18 (−2.94, +2.58)	−8.08 (−12.99, −3.17)	−7.90 (−13.47, −2.33)	0.0117 (0.0156)	0.0009
All-adult W-SRI ≥ 2	+0.39 (−1.67, +2.45)	−7.16 (−12.50, −1.81)	−7.55 (−13.84, −1.25)	0.0248 (0.0248)	0.0019

Panel B. Secondary employed-worker cumulative outcome					
Outcome	Higher anchoring (SRA 3–5), difference (95% CI)	Lower anchoring (SRA 0–2), difference (95% CI)	Between-stratum difference (95% CI)	CR2 p (BH q)	Wild bootstrap p
Employed-worker cumulative burden ≥2	+0.44 (−1.73, +2.62)	−9.57 (−13.95, −5.20)	−10.02 (−14.78, −5.25)	0.0014 (—)	—

Estimates compare the focal high-AIOE/high-task-detachability profile with lower-AIOE occupations within the stated anchoring stratum. Binary estimates are percentage point differences. The between-stratum estimate is the lower-anchoring contrast minus the higher-anchoring contrast. CR2 confidence intervals use coefficient-specific Satterthwaite degrees of freedom. Parenthetical q values are Benjamini–Hochberg adjustments of the four primary CR2 *p*-values. Wild cluster bootstrap tests for the four Panel A outcomes used 9,999 restricted null-imposed replications with Webb six-point weights. Wild cluster bootstrap inference was not calculated for the secondary employed-worker burden measure.

**Figure 2:**
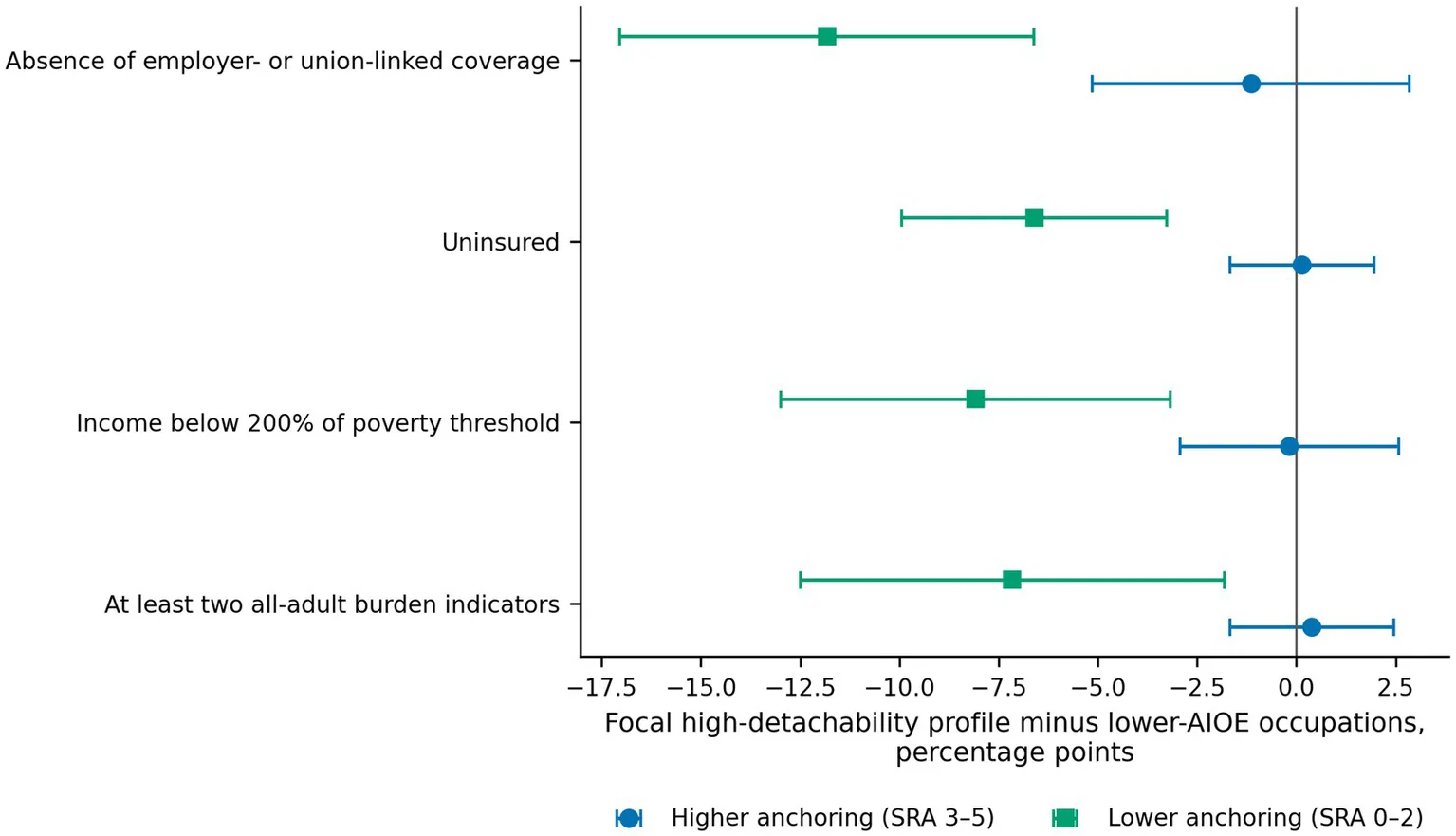
Adjusted focal occupational-profile contrasts by migrant anchoring.Note. Points show adjusted differences from lower-AIOE occupations; bars show CR2 95% confidence intervals. Negative values indicate a lower adverse-outcome prevalence in the focal profile. Models adjust for year, age, sex, race/ethnicity, education, region, and SOC major group.

The largest between-stratum difference was observed for absence of employer- or union-linked coverage (−10.68 percentage points). Differences were also evident for uninsurance, poverty, and all-adult cumulative burden (Table 2). All four between-stratum estimates had CR2 confidence intervals below zero, wild cluster bootstrap *p*-values below 0.002, and Benjamini–Hochberg q values below 0.025.

The employed-worker burden measure showed a similar separation between anchoring strata: the focal occupational profile and lower-AIOE occupations differed little among the higher-anchoring stratum, whereas cumulative burden was lower for the focal profile in the lower-anchoring stratum (Table 2). The two burden measures use different populations and components and are interpreted separately.

Across the targeted robustness specifications, the between-stratum modifications ranged from −10.61 to −10.82 percentage points for absence of employer- or union-linked coverage, −6.74 to −7.00 for uninsurance, −7.78 to −8.47 for poverty, and −7.43 to −7.94 for all-adult cumulative burden. All four CR2 confidence intervals remained below zero across the temporal, geographic, and employment status specifications reported in Supplementary Table S3A. The consistency across specifications indicates limited sensitivity to the tested temporal, geographic, and employment-status restrictions, although residual confounding remains possible.

### Labour attachment, earnings, and mobility

After restricting unemployment to respondents in the labour force, the high-AIOE/high-task-detachability versus lower-AIOE contrast was −0.28 percentage points (95% CI −0.95 to +0.39) among higher-anchoring adults and −0.65 (95% CI −1.55 to +0.25) among lower-anchoring adults. The lower-minus-higher-anchoring modification was −0.37 percentage points (95% CI −1.15 to +0.42; CR2 *p* = 0.3126). Excluding 2020 yielded −0.32 percentage points (95% CI −0.98 to +0.34; *p* = 0.2923). These estimates were imprecise, and their intervals included modest negative and positive differences. Modification estimates for non-employment and mobility were also imprecise. Annual wage contrasts were positive within both anchoring strata, but the between-stratum difference was not statistically precise; the 2019–2024 hourly wage proxy showed the same limitation (Table 3). The secondary outcome estimates were imprecise, with confidence intervals spanning both negative and positive differences.

**Table 3:** Adjusted focal occupational profile contrasts by migrant anchoring for labour attachment, earnings, and mobility.

Panel A. Secondary labour market and mobility outcomes				
Outcome	Higher anchoring (SRA 3–5), difference (95% CI)	Lower anchoring (SRA 0–2), difference (95% CI)	Between-stratum difference (95% CI)	CR2 p
Unemployment	−0.28 (−0.95, +0.39)	−0.65 (−1.55, +0.25)	−0.37 (−1.15, +0.42)	0.3126
Non-employment	−1.34 (−2.91, +0.24)	−1.02 (−5.00, +2.95)	+0.32 (−4.26, +4.89)	0.8765
Real annual wage	+13.21% (−1.19%, +29.70%)	+24.96% (+6.65%, +46.42%)	+10.39% (−1.18%, +23.31%)	0.0733
Moved within state	−0.22 (−1.09, +0.66)	+0.22 (−1.17, +1.60)	+0.44 (−1.37, +2.24)	0.5900
Abroad 1 year earlier	−0.06 (−0.78, +0.66)	+0.58 (−2.20, +3.37)	+0.64 (−2.76, +4.05)	0.6729

Panel B. Hourly wage proxy sensitivity, 2019–2024				
Outcome	Higher anchoring (SRA 3–5), difference	Lower anchoring (SRA 0–2), difference	Between-stratum difference (95% CI)	CR2 p
Hourly wage proxy, 2019–2024	+11.56% (+1.20%, +22.98%)	+19.39% (+6.12%, +34.32%)	+7.02% (−4.04%, +19.35%)	0.1884

Binary estimates are percentage point differences; wage estimates are percentage differences transformed from log wage coefficients. Unemployment is restricted to respondents in the labour force. *p*-values correspond to the displayed focal profile lower-minus-higher-anchoring estimate. The hourly wage proxy is restricted to 2019–2024 because weeks worked were consistently available only in those years.

### SRA validation

Between-stratum estimates remained negative when lower anchoring was defined as SRA5 scores of 0–1, 0–2, or 0–3, with smaller magnitudes under the broader thresholds (Supplementary Table S2A). Continuous models showed that the high-detachability contrast became progressively less negative as the anchoring score increased (Supplementary Table S2B). The contrast was most pronounced at SRA5 scores 1 and 2, smaller at score 3, and close to zero at scores 4 and 5 (Figure 3). The gradient was not confined to the binary cut point: the focal profile contrast weakened progressively as additional anchoring resources accumulated. This pattern supports interpreting SRA as an ordered contemporaneous stratification measure rather than as a single dichotomous status. Leave-one-component-out estimates were similar across exclusions of citizenship, residence duration, language, family co-residence, and homeownership (Supplementary Table S2C).

**Figure 3:**
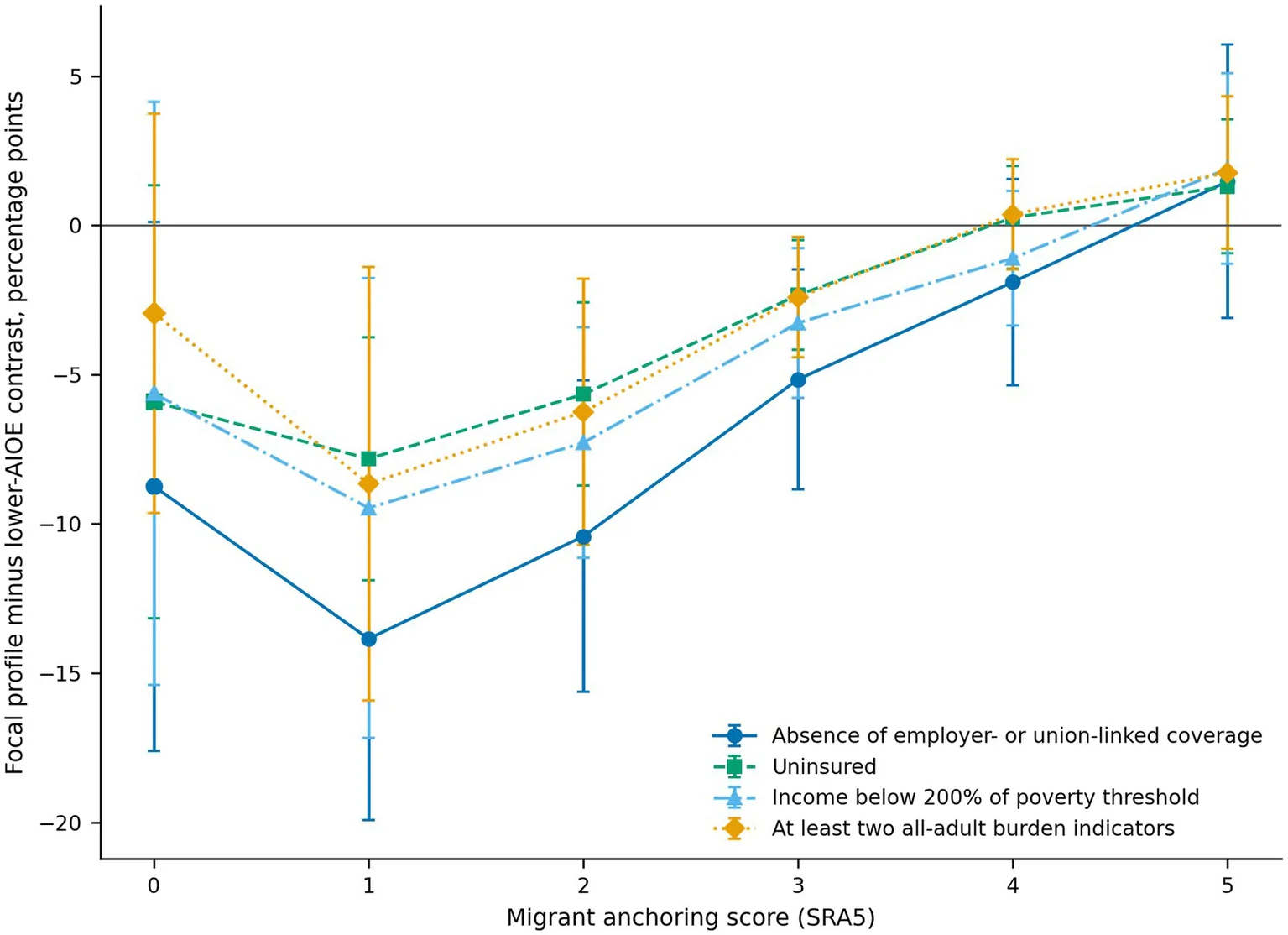
Adjusted focal occupational-profile contrasts across migrant anchoring scores.Note. Points show adjusted focal-profile minus lower-AIOE contrasts at SRA5 scores 0–5; bars show CR2 95% confidence intervals. Lines aid interpretation and do not impose a linear trend. Open markers at score 0 indicate the smallest anchoring subgroup and should be interpreted cautiously.

### Exposure definition and occupational cluster sensitivity

Occupational support by exposure category is reported in Table 1B, Panel B. Exact SOC and top tercile analyses produced negative focal between-stratum estimates for all four outcomes, although exact matching attenuated the poverty and cumulative burden differences (Supplementary Table S3B). No primary estimate changed direction when any one SOC major group was removed (Supplementary Figure S1).

The two high-detachability quadrants produced broadly similar point estimates, but direct and factorial comparisons were imprecise and did not detect a clear shielding-dependent difference (Supplementary Tables S4A,B). The wide confidence intervals precluded conclusions about statistical equivalence or the independent role of shielding.

Continuous models examined related occupational features rather than independent interventions. As AIOE, detachability, and shielding were correlated, joint coefficients were interpreted only as conditional associations. In joint models, AIOE showed the clearest associations with insurance outcomes and detachability with poverty and cumulative burden, while shielding showed no consistent cross-outcome pattern (Figure 4).

**Figure 4:**
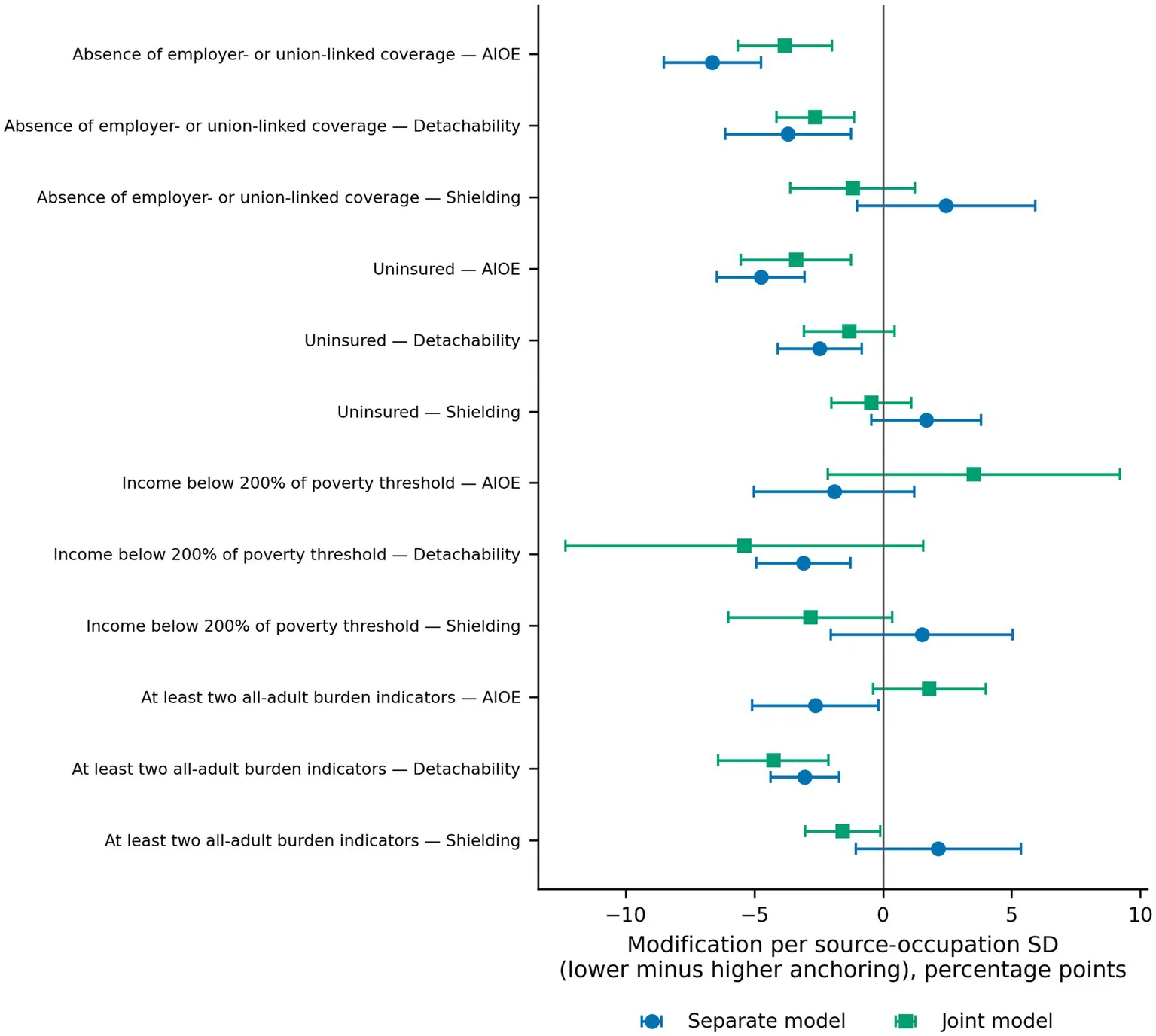
Continuous AIOE, task detachability, and shielding associations across migrant anchoring.Note. Estimates are lower-minus-higher-anchoring modifications per source-occupation standard deviation; bars show CR2 95% confidence intervals. Separate one-score models are compared with a joint model containing all three scores. Across 760 source occupations, the correlations were AIOE–detachability r = 0.7280, AIOE–shielding r = −0.3254, and detachability–shielding r = −0.5771. Joint-model coefficients are conditional and should not be interpreted as independent effects.

## Discussion

### Principal findings

The principal finding was that the association between the compound high-AIOE/high-task-detachability occupational profile and upstream insurance and material conditions differed by migrant anchoring. Among higher-anchoring adults, the focal profile and lower-AIOE occupations had similar adjusted outcome profiles; among lower-anchoring adults, the focal profile was associated with lower prevalence of absence of employer- or union-linked coverage, uninsurance, poverty, and cumulative burden. Magnitudes were similar across the outcome-specific analytic universes, exclusion of 2020, the 2018–2024 restriction, state fixed effects, and the currently employed restriction. The labour force-restricted unemployment modification and the other secondary labour market modifications were imprecisely estimated, with confidence intervals spanning negative and positive values. Taken together, the results point to context-dependent occupational stratification rather than a uniform high-AIOE advantage. The same occupational profile was associated with different levels of insurance and material protection depending on respondents’ contemporaneous anchoring resources.

### Interpreting the compound occupational profile

The focal contrast represents a compound high-AIOE/high-task-detachability occupational profile. The exposure groups differ jointly in AIOE classification, task organisation, and detailed occupational composition. Accordingly, the estimate reflects the association of the combined occupational profile with the outcomes. The two shielding contexts yielded broadly similar point estimates, but the direct and factorial comparisons had wide confidence intervals and low effective degrees of freedom. The shielding comparisons were imprecise and did not establish statistical equivalence. Continuous score coefficients were interpreted as conditional associations among correlated occupational measures.

### Migrant occupational sorting and access to coverage

The observed pattern is consistent with occupational and migration-related sorting across employment channels. One plausible interpretation is that some lower-anchoring migrants enter the United States through specialised recruitment channels that place them in professional or technical positions before they acquire long residence, citizenship, or homeownership. Their lower SRA scores may therefore coexist with English proficiency, recognised credentials, and access to benefit-bearing employment (18, 19, 24). High-AIOE/high-task-detachability occupations include professional, technical, analytical, and digitally tradable work that may be reached through specialised credentials, recruitment networks, or internationally mobile labour channels (18, 19, 24, 36, 44). Lower-anchoring degree holders in lower-AIOE occupations may face different credential recognition, occupational mobility, or employment security conditions. Coverage could derive from the respondent’s current or former employment, a union, or another eligible family member. Employer recruitment, visa-linked employment, and the respondent’s own benefit offer are therefore plausible but untested explanations.

The wage results offer limited discrimination among these potential mechanisms. Access to employer- or union-linked coverage can vary with employer size, industry, sector, full-time status, class of worker, union arrangements, and household employment, even at similar wages (12, 16). These pathways remain unresolved in the available data.

### Upstream public health meaning

The outcomes capture upstream insurance and material conditions relevant to worker health. Insurance access can affect the affordability and continuity of care, while poverty and overlapping material burden shape resources for housing, food, transport, recovery, and care (12–14, 23, 45–51). The study contributes an occupational population health perspective by showing how potential AI exposure and task organisation co-occur with access to protection across migrant-anchoring profiles. Taken together, these outcomes locate occupational health partly in the institutional resources surrounding work. They show how occupational position may be linked to the practical capacity to obtain care, absorb financial shocks, and maintain everyday living conditions. Although employer-linked insurance is specific to the United States, the underlying institutional question is broader. In other health systems, comparable forms of work-linked protection may include social insurance registration, contribution continuity, paid leave, unemployment protection, or portability of benefits across employers and migration statuses.

### Geographic context

The spatial precarity literature cautions that labour market institutions and employment quality are geographically uneven (15, 26, 27). Replacing census region with state fixed effects left the principal estimates close to the primary results, including when 2020 was excluded. Similar estimates under state fixed effects indicate limited sensitivity to broad geographic adjustment, although residual place-based confounding may remain. State policies, metropolitan labour markets, local industry composition, and employer geography may still contribute to the observed associations, and the four-region primary adjustment cannot represent those processes in detail.

### Anchoring profiles and cumulative burden

Lower anchoring did not represent a single migrant experience. The same SRA score reflected different combinations of language, citizenship, residence duration, family, and housing resources across occupational groups. Lower-anchoring adults in the focal profile were especially likely to meet the language component but rarely met the citizenship, residence duration, or homeownership components. Threshold, continuous, factor, and leave-one-component-out analyses indicated that no single SRA component generated the primary result. The two cumulative measures also showed the same broad anchoring-related pattern, but they use different universes and components. The domain-specific insurance and poverty outcomes remain more directly interpretable (Supplementary Table S5A).

### Limitations and future research

Several limitations constrain interpretation. First, repeated cross-sectional ACS data cannot establish temporal order among anchoring, occupational placement, and work-linked protection. The same factors may shape both SRA and occupation, and occupational position may influence homeownership, family arrangements, or settlement. Second, AIOE and O*NET characterise occupation-level potential and context. They do not observe individual AI use, employer adoption, implementation purpose, task reallocation, or a health response to deployment. The O*NET 30.3 task snapshot postdates the 2017–2024 ACS observations and was applied retrospectively, so changes in time-varying occupational tasks were not measured.

Third, the focal contrast represents a compound high-AIOE/high-task-detachability occupational profile rather than an independent detachability effect. Residual differences in detailed occupations can contribute to the estimates. The shielding comparisons were imprecise and did not establish equivalence. Fourth, the employer- or union-linked coverage outcome identifies access from any eligible source, not the policyholder or whether the respondent’s current job offered the policy. Interpretation in terms of the respondent’s employer, recruitment channel, or job benefits is therefore necessarily tentative.

Fifth, the focal profile was represented in only nine SOC major groups, and coefficient-specific Satterthwaite degrees of freedom were approximately 7.6–7.7 for the principal interaction terms. CR2, wild cluster bootstrap, and cluster deletion analyses address small-cluster uncertainty but cannot replace missing occupational information. ACS replicate weights were unavailable in the analytic file, so uncertainty estimates do not incorporate the complete survey replicate-weight design.

Sixth, prefix-based occupational linkage was required for 47.05% of the weighted bachelor’s-degree-or-higher source sample. The pooled crosswalk spanned the 2017 SOC vintage change, and the fixed O*NET release used a later occupational taxonomy. Exact SOC and 2018–2024 analyses retained the direction of the results, but exact matching attenuated the poverty and cumulative burden estimates, indicating meaningful sensitivity to occupational linkage. Median-based task categories also simplify continuous constructs and leave some sparse categories.

Seventh, state fixed effects did not materially change the focal estimates, but they do not address metropolitan context, time-varying state policy, or all place-based labour market conditions. Industry, class of worker, weeks worked, hours worked, visa or admission pathway, employer size, sector, benefit policy, and household policyholder were unavailable or omitted and may explain part of the associations. Eighth, valid occupation linkage requires a reported recent occupation; outcome universes differ, with the coverage outcome limited to employed wage and salary workers and unemployment limited to the labour force. Annual wage combines wage rates, weeks worked, and hours worked, and the hourly proxy was limited to 2019–2024.

Finally, multiple outcomes, exposure categories, and sensitivity analyses raise the possibility of chance findings. The four focal modifications remained supported after false discovery rate adjustment and targeted robustness checks, but exploratory category-specific, continuous, and shielding results require restraint. Future longitudinal or quasi-experimental research should link occupational potential to observed employer adoption and worker-level use and measure industry, hours worked, class of worker, visa pathway, employer attributes, benefit source, and direct health outcomes.

### Implications for sustainable digital workplaces

Occupational health monitoring should combine potential AI-task overlap with measures of task organisation, benefit continuity, material security, autonomy, work intensity, and worker voice. Operationally, this requires linking workforce technology assessments to benefit enrolment, coverage continuity, and material security indicators at the organisational level. Such monitoring could identify whether technological reorganisation coincides with gaps during recruitment, contract change, household transition, or movement between employers. The findings also draw attention to lower-AIOE occupations in which lower-anchoring adults experienced greater insurance insecurity and poverty. Employers, regulators, credentialing bodies, and migration authorities should examine whether occupational and coverage pathways create avoidable gaps during job, contract, or household transitions (14, 21, 22, 52–54). These implications support surveillance and organisational assessment of digital workplace conditions.

## Conclusion

Among highly educated foreign-born adults, a compound high-AIOE/high-task-detachability occupational profile was associated with lower prevalence of absence of employer- or union-linked coverage, uninsurance, poverty, and cumulative burden than lower-AIOE occupations among lower-anchoring adults, but not clearly among higher-anchoring adults. The estimates remained similar after excluding 2020, restricting the sample to 2018–2024, and adding state fixed effects. Secondary labour market modifications were imprecisely estimated, with confidence intervals spanning negative and positive differences. The focal comparison reflects a compound occupational profile rather than an independent effect of task detachability. The pattern is best understood as occupational stratification in work-linked protection that varies by contemporaneously observed migrant anchoring. Longitudinal and employer-linked studies are needed to distinguish occupational selection from changes that follow technological adoption.

## Data Availability

Publicly available datasets were analyzed in this study. The population microdata are available from IPUMS USA, Version 16.0: https://doi.org/10.18128/D010.V16.0. Access requires IPUMS user registration and compliance with IPUMS data-use terms. The occupation-level AI exposure data are available from the AIOE GitHub repository: https://github.com/AIOE-Data/AIOE. The occupation-level work-context and task data are available from the *ONET Resource Center*/ONET Database: https://www.onetcenter.org/database.html. CPI-U data used for wage deflation are available from the U.S. Bureau of Labor Statistics Consumer Price Index database, series CUUR0000SA0: https://data.bls.gov/timeseries/CUUR0000SA0. Analysis code, non-identifying aggregated model cells, the occupation-exposure crosswalk, data dictionaries, source-data workbook, checksum manifest, and machine-readable table and figure source data are provided in Supplementary Data 1. Individual-level IPUMS ACS microdata are not redistributed and remain subject to IPUMS registration and data-use terms. No accession numbers are applicable for these public datasets.

## References

[1] Kellogg KC Valentine MA Christin A. Algorithms at work: the new contested terrain of control. Acad Manag Ann. (2020) 14:366–410. doi: 10.5465/annals.2018.0174

[2] International Labour Organization. Revolutionizing Health and Safety: The role of AI and Digitalization at work. Geneva: International Labour Organization (2025).

[3] European Agency for Safety and Health at Work. OSH and the Future of Work: Benefits and Risks of Artificial Intelligence Tools in Workplaces. Bilbao: EU-OSHA (2019).

[4] Howard J. Artificial intelligence: implications for the future of work. Am J Ind Med. (2019) 62:917–26. doi: 10.1002/ajim.23037, 31436850

[5] Parker SK Grote G. Automation, algorithms, and beyond: why work design matters more than ever in a digital world. Appl Psychol. (2022) 71:1171–204. doi: 10.1111/apps.12241

[6] Autor DH Levy F Murnane RJ. The skill content of recent technological change: an empirical exploration. Q J Econ. (2003) 118:1279–333. doi: 10.1162/003355303322552801

[7] Arntz M Gregory T Zierahn U. The Risk of Automation for Jobs in OECD Countries: A Comparative Analysis. OECD Social, Employment and Migration Working Papers No. 189. Paris: OECD Publishing (2016).

[8] Brynjolfsson E Mitchell T Rock D. What can machines learn, and what does it mean for occupations and the economy? AEA Pap Proc. (2018) 108:43–7. doi: 10.1257/pandp.20181019

[9] Eloundou T Manning S Mishkin P Rock D. GPTs are GPTs: labor market impact potential of LLMs. Science. (2024) 384:1306–8. doi: 10.1126/science.adj0998, 38900883

[10] Gmyrek P Berg J Kaminski K Konopczynski F Ładna A Nafradi B . Generative AI and Jobs: A Refined Global Index of Occupational Exposure. ILO Working Paper 140. Geneva: International Labour Organization (2025). doi: 10.54394/HETP0387

[11] Felten EW Raj M Seamans R. Occupational, industry, and geographic exposure to artificial intelligence: a novel dataset and its potential uses. Strateg Manag J. (2021) 42:2195–217. doi: 10.1002/smj.3286

[12] Buchmueller TC Lo Sasso AT Lurie I Dolfin S. Immigrants and employer-sponsored health insurance. Health Serv Res. (2007) 42:286–310. doi: 10.1111/j.1475-6773.2006.00600.x, 17355593 PMC1955235

[13] Benach J Vives A Amable M Vanroelen C Tarafa G Muntaner C. Precarious employment: understanding an emerging social determinant of health. Annu Rev Public Health. (2014) 35:229–53. doi: 10.1146/annurev-publhealth-032013-182500, 24641559

[14] Ahonen EQ Fujishiro K Cunningham T Flynn M. Work as an inclusive part of population health inequities research and prevention. Am J Public Health. (2018) 108:306–11. doi: 10.2105/AJPH.2017.304214, 29345994 PMC5803801

[15] Oddo VM Zhuang CC Andrea SB Eisenberg-Guyot J Peckham T Jacoby D . Changes in precarious employment in the United States: a longitudinal analysis. Scand J Work Environ Health. (2021) 47:171–80. doi: 10.5271/sjweh.3939, 33283874 PMC8126438

[16] U.S. Bureau of Labor Statistics. Employee Benefits in the United States, March 2024. Washington, DC: U.S. Bureau of Labor Statistics (2024).

[17] Quesada J Hart LK Bourgois P. Structural vulnerability and health: Latino migrant laborers in the United States. Med Anthropol. (2011) 30:339–62. doi: 10.1080/01459740.2011.576725, 21777121 PMC3146033

[18] Xiang B Lindquist J. Migration infrastructure. Int Migr Rev. (2014) 48(Suppl 1)–S148. doi: 10.1111/imre.12141, 40046247

[19] Liu-Farrer G Yeoh BSA Baas M. Social construction of skill: an analytical approach toward the question of skill in cross-border labour mobilities. J Ethn Migr Stud. (2021) 47:2237–51. doi: 10.1080/1369183X.2020.1731983

[20] Castañeda H Holmes SM Madrigal DS Young MED Beyeler N Quesada J. Immigration as a social determinant of health. Annu Rev Public Health. (2015) 36:375–92. doi: 10.1146/annurev-publhealth-032013-182419, 25494053

[21] Moyce SC Schenker M. Migrant workers and their occupational health and safety. Annu Rev Public Health. (2018) 39:351–65. doi: 10.1146/annurev-publhealth-040617-013714 29400993

[22] Ahonen EQ Benavides FG Benach J. Immigrant populations, work and health: a systematic literature review. Scand J Work Environ Health. (2007) 33:96–104. doi: 10.5271/sjweh.1112, 17460797

[23] Derose KP Escarce JJ Lurie N. Immigrants and health care: sources of vulnerability. Health Aff. (2007) 26:1258–68. doi: 10.1377/hlthaff.26.5.1258, 17848435

[24] Mattoo A Neagu IC Özden Ç. Brain waste? Educated immigrants in the US labor market. J Dev Econ. (2008) 87:255–69. doi: 10.1016/j.jdeveco.2007.05.001

[25] Nwankwo E Pastor M. When the color line meets the borderline: health insurance coverage among black immigrant adults in the US. Health Aff (Millwood). (2025) 44:296–303. doi: 10.1377/hlthaff.2024.00672, 40030113

[26] Volchik V Maslyukova E Strielkowski W. Spatial effects of informal employment and precarization in urban centres and regions. Theor Empir Res Urban Manag. (2020) 15:21–37.

[27] Kapitsinis N Gialis S. The spatial division of precarious labour across the European Union regions: a composite index analysis of the 2008/2009 global economic crisis effects and COVID-19 initial implications. Eur Urban Reg Stud. (2023) 30:380–403. doi: 10.1177/09697764231191631

[28] Kofman E Raghuram P. Gendered Migrations and Global Social Reproduction. London: Palgrave Macmillan (2015).

[29] Parreñas RS. Servants of Globalization: Women, Migration, and Domestic Work. Stanford: Stanford University Press (2001).

[30] Acemoglu D Restrepo P. Robots and jobs: evidence from US labor markets. J Polit Econ. (2020) 128:2188–244. doi: 10.1086/705716

[31] Frey CB Osborne MA. The future of employment: how susceptible are jobs to computerisation? Technol Forecast Soc Change. (2017) 114:254–80. doi: 10.1016/j.techfore.2016.08.019

[32] Webb M. The Impact of Artificial Intelligence on the Labor Market.Stanford University (2020). doi: 10.2139/ssrn.3482150

[33] Wang B Liu Y Qian J Parker SK. Achieving effective remote working during the COVID-19 pandemic: a work design perspective. Appl Psychol. (2021) 70:16–59. doi: 10.1111/apps.12290, 33230359 PMC7675760

[34] Goldman N Pebley AR Lee K Andrasfay T Pratt B. Racial and ethnic differentials in COVID-19-related job exposures by occupational standing in the US. PLoS One. (2021) 16:e0256085. doi: 10.1371/journal.pone.0256085, 34469440 PMC8409606

[35] Wei CF Lan FY Hsu YT Lowery N Dibona L Akkeh R . Risk of SARS-CoV-2 infection among essential workers in a community-based cohort in the United States. Front Public Health. (2022) 10:878208. doi: 10.3389/fpubh.2022.878208, 35677773 PMC9169416

[36] Dingel JI Neiman B. How many jobs can be done at home? J Public Econ. (2020) 189:104235. doi: 10.1016/j.jpubeco.2020.104235, 32834177 PMC7346841

[37] Lunde LK Fløvik L Christensen JO Johannessen HA Finne LB Jørgensen IL . The relationship between telework from home and employee health: a systematic review. BMC Public Health. (2022) 22:47. doi: 10.1186/s12889-021-12481-2, 34996400 PMC8741267

[38] Kim TJ von dem Knesebeck O. Is an insecure job better for health than having no job at all? A systematic review of studies investigating the health-related risks of both job insecurity and unemployment. BMC Public Health. (2015) 15:985. doi: 10.1186/s12889-015-2313-1, 26419739 PMC4589035

[39] Kind AJH Buckingham WR. Making neighborhood-disadvantage metrics accessible: the neighborhood atlas. N Engl J Med. (2018) 378:2456–8. doi: 10.1056/NEJMp1802313, 29949490 PMC6051533

[40] Ruggles S Nelson MA Sobek M Fitch CA Goeken R Hacker JD . IPUMS USA: Version 16.0 (dataset). Minneapolis: IPUMS (2025).

[41] U.S. Census Bureau. 2020 ACS 1-Year Experimental Data Release: User Note Washington, DC: U.S. Census Bureau (2021). Available online at: https://www.census.gov/programs-surveys/acs/technical-documentation/user-notes/2021-02.html

[42] National Center for O*NET Development. O*NET Database 30.3, May 2026; O*NET-SOC 2019 Taxonomy Aligned with the 2018 SOC. Washington, DC: U.S. Department of Labor, Employment and Training Administration (2026).

[43] U.S. Bureau of Labor Statistics. Consumer Price Index for All Urban Consumers (CPI-U), U.S. city Average, all Items. Washington, DC: U.S. Bureau of Labor Statistics (2026).

[44] Cazzaniga M Jaumotte F Li L Melina G Panton AJ Pizzinelli C . Gen-AI: Artificial Intelligence and the Future of Work. IMF Staff Discussion Note No. 2024/001. Washington: International Monetary Fund (2024).

[45] Kaiser Family Foundation. Key Facts on Health Coverage of Immigrants. San Francisco: KFF (2024).

[46] Commission on Social Determinants of Health. Closing the Gap in a Generation: Health Equity through Action on the Social Determinants of Health. Geneva: World Health Organization (2008). 10.1016/S0140-6736(08)61690-6 18994664

[47] CDC/ATSDR. Social Vulnerability Index. Atlanta: Agency for Toxic Substances and Disease Registry (2024).

[48] Centers for Medicare & Medicaid Services. The Accountable Health Communities Health-Related Social Needs Screening Tool. Baltimore: CMS (2017).

[49] Marmot M. Social determinants of health inequalities. Lancet. (2005) 365:1099–104. doi: 10.1016/S0140-6736(05)71146-6, 15781105

[50] Krieger N. Theories for social epidemiology in the 21st century: an ecosocial perspective. Int J Epidemiol. (2001) 30:668–77. doi: 10.1093/ije/30.4.668, 11511581

[51] Link BG Phelan J. Social conditions as fundamental causes of disease. J Health Soc Behav. (1995) Spec No.:80–94. doi: 10.2307/2626958, 7560851

[52] United Nations Statistics Division. SDG Indicator Metadata: Indicator 10.7.1, Recruitment Cost Borne by Employee as a Proportion of Monthly Income Earned in Country of Destination. New York: United Nations (2025).

[53] United Nations Statistics Division. SDG Indicator Metadata: Indicator 8.8.1, Fatal and Non-fatal Occupational Injuries per 100,000 Workers, by Sex and Migrant Status. New York: United Nations (2026).

[54] United Nations General Assembly. Global Compact for Safe, Orderly and Regular Migration. New York: United Nations (2018).

