# Supplementary material for: AI exposure and task detachability: migrant anchoring and work-linked health protection among highly educated foreign-born adults in the United States
Source: Front Public Health. 2026 Oct 5;14:1929386. doi: 10.3389/fpubh.2026.1929386 (PMC13677537; doi:10.3389/fpubh.2026.1929386)
Supplement: Supplementary file 1 [file Supplementary_file_1.DOCX]

***Supplementary Material***

*Occupation-level AI exposure and task detachability: migrant anchoring and work-linked health protection*

**Supplementary Tables S1–S5 and Supplementary Figure S1**

**Supplementary Table S1. Weighted sample characteristics and SRA component composition**

**Panel A. Analytic-sample characteristics overall and by anchoring**

| **Characteristic** | **Overall, % (n=746,995; N=83,420,333)** | **Higher anchoring, % (n=603,411; N=64,678,265)** | **Lower anchoring, % (n=143,584; N=18,742,068)** |
| --- | --- | --- | --- |
| Age: 25-34 | 24.86 | 17.58 | 49.96 |
| 35-44 | 31.30 | 31.34 | 31.15 |
| 45-54 | 26.27 | 30.12 | 12.97 |
| 55-64 | 17.58 | 20.96 | 5.91 |
| Sex: Female | 49.35 | 51.00 | 43.67 |
| Male | 50.65 | 49.00 | 56.33 |
| Race/ethnicity: American Indian/Alaska Native, non-Hispanic | 0.04 | 0.04 | 0.03 |
| Asian/Pacific Islander, non-Hispanic | 44.96 | 45.32 | 43.73 |
| Black, non-Hispanic | 9.17 | 9.41 | 8.36 |
| Hispanic | 20.17 | 18.98 | 24.27 |
| Multiracial, non-Hispanic | 2.55 | 2.47 | 2.83 |
| Other, non-Hispanic | 0.71 | 0.64 | 0.95 |
| White, non-Hispanic | 22.40 | 23.14 | 19.83 |
| Education: Bachelor | 54.35 | 54.60 | 53.48 |
| Master | 31.29 | 30.68 | 33.40 |
| Professional | 6.80 | 7.08 | 5.86 |
| Doctorate | 7.56 | 7.64 | 7.27 |
| Region: Northeast | 23.23 | 23.07 | 23.78 |
| Midwest | 12.21 | 11.88 | 13.32 |
| South | 33.78 | 33.59 | 34.43 |
| West | 30.79 | 31.46 | 28.47 |

**Note.** Percentages are weighted within the stated analytic sample or anchoring stratum. Exposure-category composition is reported in Main Table 1 and is not repeated here.

**Panel B. SRA component prevalence by exposure category and anchoring stratum**

| **Exposure category** | **anchoring stratum** | **Weighted n** | **Citizenship %** | **≥10 years residence %** | **Language anchoring %** | **Family co-residence %** | **Homeownership %** |
| --- | --- | --- | --- | --- | --- | --- | --- |
| Lower AIOE | Higher anchoring | 33,472,803 | 77.98 | 88.36 | 96.27 | 82.56 | 76.01 |
| Lower AIOE | Lower anchoring | 9,695,939 | 4.41 | 15.10 | 72.47 | 57.18 | 11.26 |
| Focal high-AIOE/high-task-detachability profile | Higher anchoring | 22,125,227 | 72.32 | 86.88 | 98.67 | 84.59 | 82.11 |
| Focal high-AIOE/high-task-detachability profile | Lower anchoring | 6,429,753 | 2.44 | 8.99 | 92.97 | 55.35 | 7.43 |
| Low detachability / high shielding | Higher anchoring | 7,796,146 | 73.87 | 88.35 | 97.98 | 84.27 | 79.11 |
| Low detachability / high shielding | Lower anchoring | 2,319,322 | 2.65 | 11.41 | 88.25 | 52.98 | 8.31 |
| Low detachability / low shielding | Higher anchoring | 649,500 | 79.51 | 87.93 | 97.77 | 78.57 | 75.08 |
| Low detachability / low shielding | Lower anchoring | 142,519 | 4.84 | 15.10 | 85.42 | 56.47 | 10.70 |
| High AIOE / task score unavailable | Higher anchoring | 634,589 | 75.42 | 87.71 | 98.58 | 81.36 | 80.13 |
| High AIOE / task score unavailable | Lower anchoring | 154,535 | 2.94 | 15.49 | 90.33 | 49.38 | 8.53 |

**Note.** Component values are weighted percentages within each exposure-by-anchoring stratum.

**Supplementary Table S2. SRA operationalisation and sensitivity analyses**

**Panel A. Alternative lower-anchoring thresholds**

| **Outcome** | **Lower anchoring definition** | **Modification, pp (95% CI)** | **CR2 p** | **df** |
| --- | --- | --- | --- | --- |
| Absence of employer- or union-linked coverage | SRA ≤1 | −11.61 (−16.61, −6.60) | 0.0009 | 7.12 |
| Absence of employer- or union-linked coverage | SRA ≤2 | −10.68 (−15.88, −5.47) | 0.0016 | 7.69 |
| Absence of employer- or union-linked coverage | SRA ≤3 | −8.81 (−13.60, −4.02) | 0.0029 | 7.90 |
| Uninsured | SRA ≤1 | −7.64 (−11.69, −3.59) | 0.0029 | 7.08 |
| Uninsured | SRA ≤2 | −6.75 (−10.57, −2.92) | 0.0039 | 7.57 |
| Uninsured | SRA ≤3 | −5.53 (−8.96, −2.10) | 0.0060 | 7.81 |
| Poverty below 200% | SRA ≤1 | −7.84 (−15.97, +0.29) | 0.0566 | 7.08 |
| Poverty below 200% | SRA ≤2 | −7.90 (−13.47, −2.33) | 0.0117 | 7.57 |
| Poverty below 200% | SRA ≤3 | −6.59 (−10.76, −2.41) | 0.0067 | 7.81 |
| All-adult W-SRI ≥2 | SRA ≤1 | −8.00 (−15.82, −0.18) | 0.0462 | 7.08 |
| All-adult W-SRI ≥2 | SRA ≤2 | −7.55 (−13.84, −1.25) | 0.0248 | 7.57 |
| All-adult W-SRI ≥2 | SRA ≤3 | −6.19 (−10.93, −1.44) | 0.0170 | 7.81 |

**Note.** The same thresholds were applied without selection based on statistical significance.

**Panel B. Continuous SRA5 interactions**

| **Outcome** | **Change in focal occupational-profile contrast per SRA point, pp (95% CI)** | **CR2 p** | **df** |
| --- | --- | --- | --- |
| Absence of employer- or union-linked coverage | +3.85 (+1.85, +5.85) | 0.0022 | 7.89 |
| Uninsured | +2.38 (+0.94, +3.82) | 0.0053 | 7.83 |
| Poverty below 200% | +2.88 (+1.00, +4.76) | 0.0077 | 7.83 |
| All-adult W-SRI ≥2 | +2.62 (+0.60, +4.64) | 0.0173 | 7.83 |

**Note.** Positive values indicate that the focal occupational-profile contrast becomes less negative as SRA5 increases.

**Supplementary Table S2 (continued)**

**Panel C. Leave-one-SRA-component-out continuous interactions**

| **Outcome** | **Excluded component** | **Change across full normalised retained score, pp (95% CI)** | **CR2 p** | **df** |
| --- | --- | --- | --- | --- |
| Absence of employer- or union-linked coverage | Citizenship | +19.11 (+9.45, +28.78) | 0.0019 | 7.82 |
| Absence of employer- or union-linked coverage | Residence duration | +18.54 (+8.64, +28.44) | 0.0026 | 7.87 |
| Absence of employer- or union-linked coverage | Language | +14.68 (+6.72, +22.65) | 0.0028 | 7.99 |
| Absence of employer- or union-linked coverage | Family co-residence | +18.40 (+8.98, +27.81) | 0.0021 | 7.80 |
| Absence of employer- or union-linked coverage | Homeownership | +16.30 (+7.73, +24.87) | 0.0024 | 7.85 |
| Uninsured | Citizenship | +10.46 (+3.92, +17.00) | 0.0063 | 7.76 |
| Uninsured | Residence duration | +12.27 (+4.58, +19.96) | 0.0064 | 7.81 |
| Uninsured | Language | +9.67 (+3.71, +15.63) | 0.0057 | 7.94 |
| Uninsured | Family co-residence | +10.30 (+4.18, +16.42) | 0.0048 | 7.74 |
| Uninsured | Homeownership | +11.02 (+4.48, +17.56) | 0.0048 | 7.78 |
| Poverty below 200% | Citizenship | +14.98 (+5.08, +24.89) | 0.0084 | 7.76 |
| Poverty below 200% | Residence duration | +14.13 (+4.95, +23.31) | 0.0077 | 7.81 |
| Poverty below 200% | Language | +12.09 (+4.19, +19.98) | 0.0078 | 7.94 |
| Poverty below 200% | Family co-residence | +12.61 (+4.49, +20.72) | 0.0073 | 7.74 |
| Poverty below 200% | Homeownership | +11.19 (+2.99, +19.39) | 0.0138 | 7.78 |
| All-adult W-SRI ≥2 | Citizenship | +12.34 (+2.60, +22.08) | 0.0194 | 7.76 |
| All-adult W-SRI ≥2 | Residence duration | +12.33 (+3.14, +21.52) | 0.0150 | 7.81 |
| All-adult W-SRI ≥2 | Language | +11.45 (+3.04, +19.86) | 0.0138 | 7.94 |
| All-adult W-SRI ≥2 | Family co-residence | +11.23 (+1.82, +20.65) | 0.0252 | 7.74 |
| All-adult W-SRI ≥2 | Homeownership | +11.47 (+2.13, +20.81) | 0.0223 | 7.78 |

**Note.** Each score is the proportion of the four retained anchoring domains present.

**Supplementary Table S3. Robustness of the focal occupational-profile estimates**

**Panel A. Temporal, geographic and employment-status specifications**

| **Specification** | **Absence of employer/union-linked coverage** | **Uninsured** | **Poverty <200%** | **All-adult W-SRI ≥2** |
| --- | --- | --- | --- | --- |
| Corrected all years | −10.68 (−15.88, −5.47); p=0.0016 | −6.75 (−10.57, −2.92); p=0.0039 | −7.90 (−13.47, −2.33); p=0.0117 | −7.55 (−13.84, −1.25); p=0.0248 |
| Exclude 2020 | −10.82 (−16.04, −5.59); p=0.0015 | −6.93 (−10.83, −3.04); p=0.0037 | −7.91 (−13.51, −2.31); p=0.0119 | −7.68 (−13.94, −1.42); p=0.0225 |
| 2018–2024 only | −10.67 (−15.88, −5.46); p=0.0016 | −6.74 (−10.58, −2.90); p=0.0039 | −7.78 (−13.32, −2.24); p=0.0121 | −7.43 (−13.58, −1.29); p=0.0238 |
| State fixed effects | −10.61 (−15.73, −5.49); p=0.0015 | −6.79 (−10.66, −2.92); p=0.0039 | −7.89 (−13.44, −2.33); p=0.0116 | −7.56 (−13.91, −1.21); p=0.0255 |
| State FE + exclude 2020 | −10.76 (−15.91, −5.61); p=0.0014 | −6.97 (−10.90, −3.03); p=0.0038 | −7.88 (−13.47, −2.30); p=0.0119 | −7.69 (−14.01, −1.37); p=0.0233 |
| Currently employed | −10.68 (−15.88, −5.47); p=0.0016 | −7.00 (−10.84, −3.15); p=0.0032 | −8.47 (−13.17, −3.77); p=0.0034 | −7.94 (−11.82, −4.07); p=0.0017 |

**Note.** Entries are lower-minus-higher-anchoring modifications, in percentage points with CR2 95% confidence intervals and coefficient-specific p values. State fixed-effects models replace census-region indicators. These are targeted robustness specifications rather than new primary estimands.

**Panel B. Exposure linkage, high-AIOE threshold and occupational influence**

| **Outcome** | **Q75 all links, CR2 pp (95% CI)** | **Q75 exact SOC, CR2 pp (95% CI)** | **Top tercile, CR2 pp (95% CI)** | **Leave-one-SOC-major range, pp** | **Sign changed** |
| --- | --- | --- | --- | --- | --- |
| Absence of employer- or union-linked coverage | −10.68 (−15.88, −5.47) | −10.16 (−14.12, −6.20) | −11.22 (−17.41, −5.02) | −12.10 to −9.09 | No |
| Uninsured | −6.75 (−10.57, −2.92) | −6.55 (−10.21, −2.90) | −7.42 (−11.67, −3.17) | −7.48 to −5.46 | No |
| Poverty below 200% | −7.90 (−13.47, −2.33) | −5.00 (−7.41, −2.58) | −8.10 (−12.87, −3.33) | −8.69 to −5.63 | No |
| All-adult W-SRI ≥2 | −7.55 (−13.84, −1.25) | −4.46 (−7.28, −1.63) | −7.61 (−12.72, −2.51) | −8.45 to −4.59 | No |

**Note.** All entries are lower-minus-higher-anchoring modifications in percentage points. Q75 and top-tercile entries include CR2 95% confidence intervals; leave-one-SOC-major entries show the full range after deleting each two-digit SOC-major group.

**Supplementary Table S4. High-AIOE task-context comparisons**

**Panel A. Direct comparison of the two high-detachability quadrants**

| **Outcome** | **Quadrant difference, pp** | **CR2 95% CI** | **CR2 p** | **df** |
| --- | --- | --- | --- | --- |
| Absence of employer- or union-linked coverage | +0.62 | −5.84 to +7.08 | 0.7887 | 3.32 |
| Uninsured | −0.61 | −5.23 to +4.01 | 0.7173 | 3.39 |
| Poverty below 200% | −0.64 | −9.01 to +7.74 | 0.8338 | 3.39 |
| All-adult W-SRI ≥2 | −1.98 | −12.25 to +8.28 | 0.6000 | 3.39 |

**Note.** The contrast is high-detachability/low-shielding minus high-detachability/high-shielding. Confidence intervals included zero; statistical equivalence was not established.

**Panel B. Shielding as a second-layer task context within high AIOE**

| **Outcome** | **Shielding-dependent difference, pp** | **CR2 95% CI** | **CR2 p** | **df** |
| --- | --- | --- | --- | --- |
| Absence of employer- or union-linked coverage | +3.32 | −5.13 to +11.77 | 0.2966 | 2.94 |
| Uninsured | +5.29 | −4.19 to +14.77 | 0.1849 | 3.43 |
| Poverty below 200% | −5.31 | −30.33 to +19.72 | 0.5686 | 3.43 |
| All-adult W-SRI ≥2 | +4.71 | −12.49 to +21.91 | 0.4690 | 3.43 |

**Note.** The three-way term tests whether shielding changes the detachability-by-anchoring pattern within high-AIOE occupations.

**Supplementary Table S5. Outcome definitions and analytic denominators**

**Panel A. Cumulative burden definitions**

| **Measure** | **Universe** | **Components** | **Threshold** | **Weighted prevalence, %** | **Interpretive note** |
| --- | --- | --- | --- | --- | --- |
| All-adult W-SRI | All linked eligible adults | Uninsurance; nonemployment; poverty below 200%; moved during the previous year | At least 2 of 4 | 9.45 | Focal cumulative summary of overlapping all-adult burden |
| Employed-worker burden | Employed wage/salary workers | Absence of employer- or union-linked coverage; poverty below 200%; moved during the previous year | At least 2 of 3 | 8.39 | Secondary marker within the employment-benefit universe |

**Note.** The all-adult and employed-worker measures use distinct universes to avoid mechanically counting nonemployment twice.

**Panel B. Outcome-specific analytic denominators**

| **Outcome** | **Unweighted person-years** | **Weighted person-years** | **Weighted events** | **Unadjusted prevalence, %** |
| --- | --- | --- | --- | --- |
| Absence of employer- or union-linked coverage | 603,484 | 67,622,356 | 11,886,124 | 17.577 |
| Uninsured | 746,995 | 83,420,333 | 6,160,168 | 7.384 |
| Poverty below 200% | 746,995 | 83,420,333 | 10,051,712 | 12.049 |
| All-adult W-SRI ≥2 | 746,995 | 83,420,333 | 7,881,344 | 9.448 |
| Unemployment (labour force) | 688,208 | 77,110,690 | 2,175,851 | 2.822 |
| Nonemployment | 746,995 | 83,420,333 | 8,485,494 | 10.172 |
| Real annual wage | 667,540 | 74,702,674 | — | — |
| Hourly-wage proxy, 2019–2024 | 515,212 | 57,871,195 | — | — |
| Moved within state | 746,995 | 83,420,333 | 14,208,735 | 17.033 |
| Abroad one year earlier | 746,995 | 83,420,333 | 2,416,437 | 2.897 |
| Employed-worker burden ≥2 | 603,484 | 67,622,356 | 5,670,993 | 8.386 |

**Note.** Weighted denominators use the ACS person weight. The hourly-wage proxy is restricted to 2019–2024. Unemployment is restricted to LABFORCE=2, with EMPSTAT=2 as the event and EMPSTAT=1 as the non-event. Event totals and prevalence are descriptive, weighted and unadjusted; wage rows are not binary outcomes.

**Supplementary Figure S1. Leave-one-SOC-major-group-out influence analysis**

**Note.** Small points show the focal occupational-profile modification after deleting each of the 22 two-digit SOC-major groups. Open diamonds show the corresponding full-sample estimate. Vertical jitter is used only to separate overlapping points. No principal estimate changed direction.
